# Gamma synuclein promotes cancer metastasis through the MKK3/6-p38MAPK cascade

**DOI:** 10.7150/ijbs.69155

**Published:** 2022-05-01

**Authors:** Jieya Liu, Ting Shao, Jin Zhang, Qianyi Liu, Hui Hua, Hongying Zhang, Jiao Wang, Ting Luo, Yuenian Eric Shi, Yangfu Jiang

**Affiliations:** 1 Laboratory of Oncogene, State Key Laboratory of Biotherapy, West China Hospital, Sichuan University, Chengdu, China; 2 Laboratory of Stem Cell Biology, West China Hospital, Sichuan University. Chengdu, China; 3 School of Basic Medicine, Chengdu University of Traditional Chinese Medicine. Chengdu, China; 4 Cancer center, West China Hospital, Sichuan University. Chengdu, China; 5 Chimigen Bio, Chengdu, China; 6 Current address: BeiGene (Shanghai) Co., Ltd, Shanghai, China

## Abstract

Gamma synuclein (SNCG) is a neuronal protein that is also aberrantly overexpressed in various types of human cancer. SNCG overexpression promotes cancer invasion and metastasis. However, the mechanisms that drive cancer metastasis upon SNCG expression remain elusive. Elucidation of the mechanisms underlying the promotion of cancer metastasis by SNCG may help discover therapeutic avenues for SNCG-overexpressed cancer. Here, we show that SNCG promotes transforming growth factor-β (TGF-β)-induced p38 mitogen-activated protein kinase (MAPK) phosphorylation. Mechanistically, SNCG promotes p38MAPK phosphorylation by interacting with the MAPK kinase 3/6 (MKK3/6) and prevents their degradation. SNCG knockdown leads to a decrease in TGF-β-induced phosphorylation of MKK3/6; and abrogates the induction of matrix metalloproteinase (MMP)-9 expression by TGF-β and its target gene Twist1. Furthermore, p38MAPK inhibition abrogates the promotion of MMP-9 expression and cancer cell invasion by SNCG. Both p38MAPK and MMP inhibitors can suppress the promotion of cancer cell invasion by SNCG. Finally, overexpression of SNCG in liver cancer cells promotes lung metastasis, which can be suppressed by the p38MAPK inhibitor. Together, our data uncover a previously unknown role of SNCG in promoting TGF-β-MKK3/6-p38MAPK signaling. This study highlights the critical role of p38MAPK in the promotion of cancer metastasis by SNCG, and indicates that p38MAPK inhibitor may serve as a potential therapeutic for SNCG-overexpressed cancer.

## Introduction

Regional lymph node spread and distant metastasis are inherent features of malignant tumors. While early-stage cancer may be effectively treated by multiple procedures such as surgical resection, radiotherapy and chemotherapy, it is still hard to treat metastatic cancer. Hence, the prognosis of patients with metastatic cancer remains to be very poor. The dissemination of cancer cells and development of organ-specific metastasis involve multiple steps, including tumor cells detachment from the primary site, migration, invasion, intravasation, survival in the circulation, extravasation, colonization at distant sites and metastatic outgrowth 1. Oncogenes, growth factors, inflammatory factors and cancer microenvironment may jointly promote cancer metastasis by enhancing cancer cell motility, invasion, colonization and angiogenesis 1-5. A better understanding the mechanisms of cancer metastasis may help uncover novel biomarkers and therapeutic targets 6.

Transforming growth factor-β (TGF-β) is a well-known growth factor that may promote cancer metastasis, stemness and immune evasion, whilst it may inhibit cell growth at the early stage of tumorigenesis 7, 8. TGF-β can induce the expression of many genes by activating mothers against decapentaplegic homolog (SMADs). Whereas the TGF-β responsive p21Cip1 and p15Ink4b may inhibit cell proliferation, Twist1, snail, ZEB1 can induce epithelial-mesenchymal transition (EMT), a process that is tightly involved in cancer metastasis 7. On the other hand, the non-SMAD pathways, such as extracellular signal-activated kinase (Erk), Jun N-terminal kinase (JNK) and p38 mitogen-activated protein kinase (p38MAPK), can be activated by TGF-β receptor signaling 9. p38MAPK is a growth factors-, cytokines- and sresses-activated kinase that has complex roles in tumorigenesis 10, 11. Among the 4 members of p38MAPK family, p38α is universially expressed in most cell types, whereas p38β, p38γ and p38δ expression are restricted in specific cell types. Whilst p38MAPK activation may inhibit the proliferation of some types of cells, it is often subverted by cancer cells to promote tumor growth, metastasis and drug resistance.

Previously, we reported that gamma synuclein (SNCG), one of three members of the synuclein family (α-synuclein, β-synuclein and SNCG) that are preferentially expressed in the nervous system, is a TGF-β responsive gene that can be induced by SMAD-mediated Twist1 expression 12. Of note, overexpression of SNCG is detected in numerous types of malignant tumors, including breast, ovary, liver, and cervical carcinoma 13-16. Previous studies have demonstrated that SNCG is more frequently upregulated in late stage of cancers, and its expression correlates with cancer metastasis and poor prognosis 13, 14, 16-19. Overexpression of SNCG in breast cancer cells promotes metastasis in a murine model of breast carcinoma 20, while SNCG knockdown suppresses perineural invasion and liver metastasis in mouse models of pancreatic carcinoma 21. Mechanistically, SNCG may promote breast and ovarian cancer cells migration by activating Erk and phosphoinositide 3-kinase (PI3K)/Akt 22-24. Moreover, secreted SNCG can enhance the motility of colorectal carcinoma cells by activating β1 integrin-focal adhesion kinase signaling 25.

While SNCG is induced by TGF-β in SMAD-dependent manner, it is unclear whether SNCG feeds back to regulate the non-SMAD pathways in TGF-β signaling, and how SNCG may mediate the pro-metastasis effect of TGF-β. In the current study, we investigate the effects of SNCG on TGF-β-induced activation of MAPK pathways. Herein, we report that SNCG promotes TGF-β-induced phosphorylation of p38MAPK by stabilizing MAPK kinase 3/6 (MKK3/6). The upregulation of p38MAPK by SNCG leads to increased MMP-9 expression, which promotes cancer cell invasion. Overexpression of SNCG in HCC cells promotes lung metastasis in mice. Treatment with p38MAPK inhibitor suppresses the promotion of HCC metastasis by SNCG.

## Materials and Methods

### Reagents

TGF-β was purchased from PeproTech (Rocky Hill, NJ, USA). The p38MAPK inhibitor SB203580 and MMP inhibitor BB-94 were from MedChemExpress (NJ, USA). The proteasome inhibitor MG132 was from Merk-Millipore (Darmstadt, Germany). Cycloheximide was from Beyotime Biotechnology (Shanghai, China). The antibodies used were as follows: anti-Twist1, anti-FLAG, anti-phosphorylated MKK3 (S189) and anti-phosphorylated MKK6 (S207) (Abcam, Cambridge, UK); anti-SNCG and anti-β-actin (Santa Cruz Biotechnology, Santa Cruz, CA, USA); anti-Erk1/2, anti-phosphorylated Erk1/2 (T202/Y204) and anti-phosphorylated p38MAPK (T180/Y182) (Cell Signaling Technology, Beverly, MA, USA); anti-p38α and anti-vimentin (Proteintech, Rosemont, IL, USA); anti-MMP-9, anti-SMAD3, anti-MKK3/6, anti-fibronection, anti-N-cadherin (Abways Technology, Shanghai, China). The pcDNA3-Twist1 and PCI-SNCG plasmids were prepared as described previously 12, 20.

### Preparation of FLAG-tagged SNCG plasmids

The C-terminal truncated SNCG expression plasmid (PCI-SNCG_∆106-127_) was constructed by mutagenesis of Gln 106 site (CAA) into the stop codon (UAA) using Mut Express II Fast Mutagenesis Kit V2 Kit (Vazyme, Nanjing, China). The primers for site-directed mutagenesis were 5′-TCTGCCCCCtAACAGGAGGGTGAGGCATCCAA-3′ (forward) and 5′-TCCTGTTaGGGGGCAGATGGCCTCAAGTCCTC-3′ (reverse). Both the full-length SNCG and truncated SNCG constructs were then cloned into pcDNA3.1-3xFlag plasmid ClonExpress II using One Step Cloning Kit (Vazyme, Nanjing, China), generating expression plasmids for FLAG-tagged SNCG (SNCG-FLAG) and truncated SNCG (SNCG_∆106-127_-FLAG). The forward PCR primer for constructing SNCG-FLAG and SNCG_∆106-127_-FLAG were 5′-AACGGGCCCTCTAGACTCGAGATGGATGTCTTCAAGAAGGGCTT-3′. The reverse primers for SNCG-FLAG and SNCG_∆106-127_-FLAG were 5′-CTTGGTACCGAGCTCGGATCCAAGTCTCCCCCACTCTGGGC-3′ and 5′-AACGGGCCCTCTAGACTCGAGATGGATGTCTTCAAGAAGGGCTT-3′, respectively.

### Cell culture

Liver cancer cell line HepG2 and cervical cancer cell line HeLa were obtained from Cell Lines Bank, Chinese Academy of Science (Shanghai, China). The cells were cultured in DMEM supplemented with 10% new born calf serum (Thermo Fisher Scientific, Waltham, MA, USA) as described 26.

### RNA interference

All siRNAs were custom-synthesized products of Ribobio Co. Ltd. (Guangzhou, China). The siR-Ribo negative control (siControl) was used for all siRNA experiments. The target sequences for SNCG and p38α knockdown are listed in Table S1. siRNA transfection was conducted as described 12.

### Western blot analysis and immunoprecipitation

Protein extracts were prepared by lysis of cells in ice-cold radioimmunoprecipitation assay (RIPA) buffer. About 30 µg of total proteins were resolved by SDS-PAGE, followed by western blotting as described 12. For immunoprecipitation, protein extracts were prepared by lysis buffer for immunoprecipitation containing 20 mM Tris (pH 7.5), 150 mM NaCl, 1% Triton X-100, 2 mM sodium phosphate, 25 mM β-glycerophosphate, 1 mM EDTA and proteinases inhibitors (Beyotime). One miligram of total proteins were incubated with primary antibodies or normal immunoglobulin G at 4°C overnight, and then incubated with 30 μl protein G agarose beads for 2 h at 4°C. The agarose-bound proteins were detected by western blotting.

### Quantitative reverse transcription (RT)-PCR analysis

The forward and reverse primers used in this study are shown in Table S2. Quantitative RT-PCRs were performed as described 26. The relative levels of target genes expression were normalized with the endogenous reference gene *GAPDH*.

### Cell migration and invasion assay

Cell migration and invasion were determined by wound-healing and transwell assays as described previously 6, 12.

### Mouse model of lung metastasis

All animal care and experiments were reviewed and approved by the Institutional Animal Care and Use Committee (IACUC) of West China Hospital of Sichuan University (approvement number: 20211268A). All animal experimentation strictly adhered to the ARRIVE guidelines and the protocol approved by IACUC of West China Hospital. Five-week-old athymic nude male mice provided by Vital River Laboratories (Beijing, China) were used in this study. To generate lung metastasis models, the empty vector- or SNCG expression plasmid-transfected HepG2 cells (2 × 10^6^/mL; 0.15 mL per mice) were injected into the tail vein. Three days later, both the control and SNCG-overexpression groups were randomly divided into groups (6-7 mice/group) that were treated with intraperitoneal injection of vehicle or SB203580 (1 mg/kg). The body weight was measured every 3 days. After 6 weeks, the mice were sacrificed, and all lungs were harvested. The lung tissues were fixed with 4% paraformaldehyde, embedded in paraffin and cut into 5 μm sections. The lung sections were then subjected to hematoxylin-eosin staining. The metastatic foci in lung sections were checked under a microscopy. The area of metastatic foci was measured by ImageJ.

### Immunohistochemical staining

The lung tissues from murine model of metastasis were fixed with 4% paraformaldehyde, embedded in paraffin, and cut into 5 µm sections. The expression of SNCG, MKK3/6 and phosphorylated p38MAPK were detected by immunohistochemical staining as described previously 10. The antibodies for SNCG (1 mg/ml), MKK3/6 (1.3 mg/ml) and phosphorylated p38MAPK (33 μg/ml) were used at 1:50, 1:200 and 1:800 dilutions, respectively. Images of the stained sections were captured under a microscope at a magnification of 400×.

### Statistical analysis

One-way analysis of variance with *post hoc* tests or Student's *t*-test (two-tailed) was used in statistical analysis. Each group size in all analysis is at least *n* = 3 (exact size is indicated in the figure legends). Data are presented as the mean ± standard deviation (SD) for *in vitro* studies or mean ± SEM for animal study. Differences are considered statistically significant if *p* < 0.05.

## Results

### SNCG knockdown reduces TGF-β-induced phosphorylation of p38 MAPK

**Figure 1:**
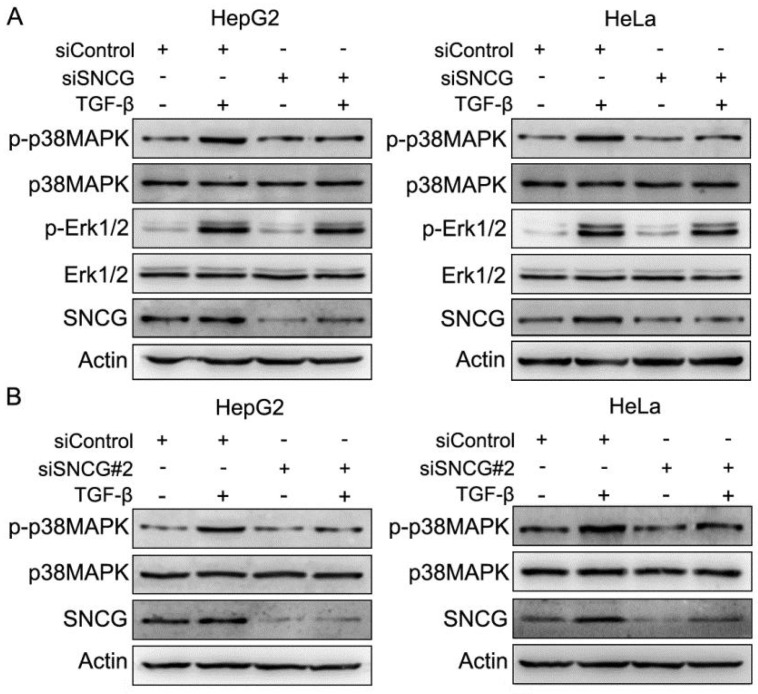
** SNCG knockdown suppresses TGF-β-induced p38MAPK phopshorylation. (A)** HepG2 and HeLa cells were transfected with siControl or siSNCG. Twenty-four hours later, the cells were treated with 5 ng/ml TGF-β for another 48h, followed by western blot analysis of indicated proteins.** (B)** HepG2 and HeLa cells were transfected with siControl or siSNCG#2. Twenty-four hours later, the cells were treated with 5 ng/ml TGF-β for another 48h, followed by western blot analysis of indicated proteins.

TGF-β may initiate SMAD and non-SMAD pathways including MAPK pathways. To determine whether SNCG regulates MAPK pathways in TGF-β signaling, we detected the effects of SNCG knockdown on TGF-β-induced p38MAPK, JNK and Erk1/2 phosphorylation. While TGF-β induced SNCG expression and p38MAPK phosphorylation, SNCG knockdown by two different sets of siRNA consistently abrogated the induction of p38MAPK phosphorylation by TGF-β in HepG2 and HeLa cells (Fig. 1). However, SNCG knockdown did not suppress the induction of Erk1/2 phosphorylation by TGF-β in both HepG2 and HeLa cells (Fig. 1). In addition, SNCG knockdown had no effect on the induction of JNK phosphorylation by TGF-β (Fig. S1). Together, these data demonstrate that SNCG is not only a TGF-β-responsive protein, but also a positive regulator of TGF-β-induced p38MAPK phosphorylation.

### SNCG stabilizes MKK3/6 to promote p38MAPK phosphorylation

**Figure 2:**
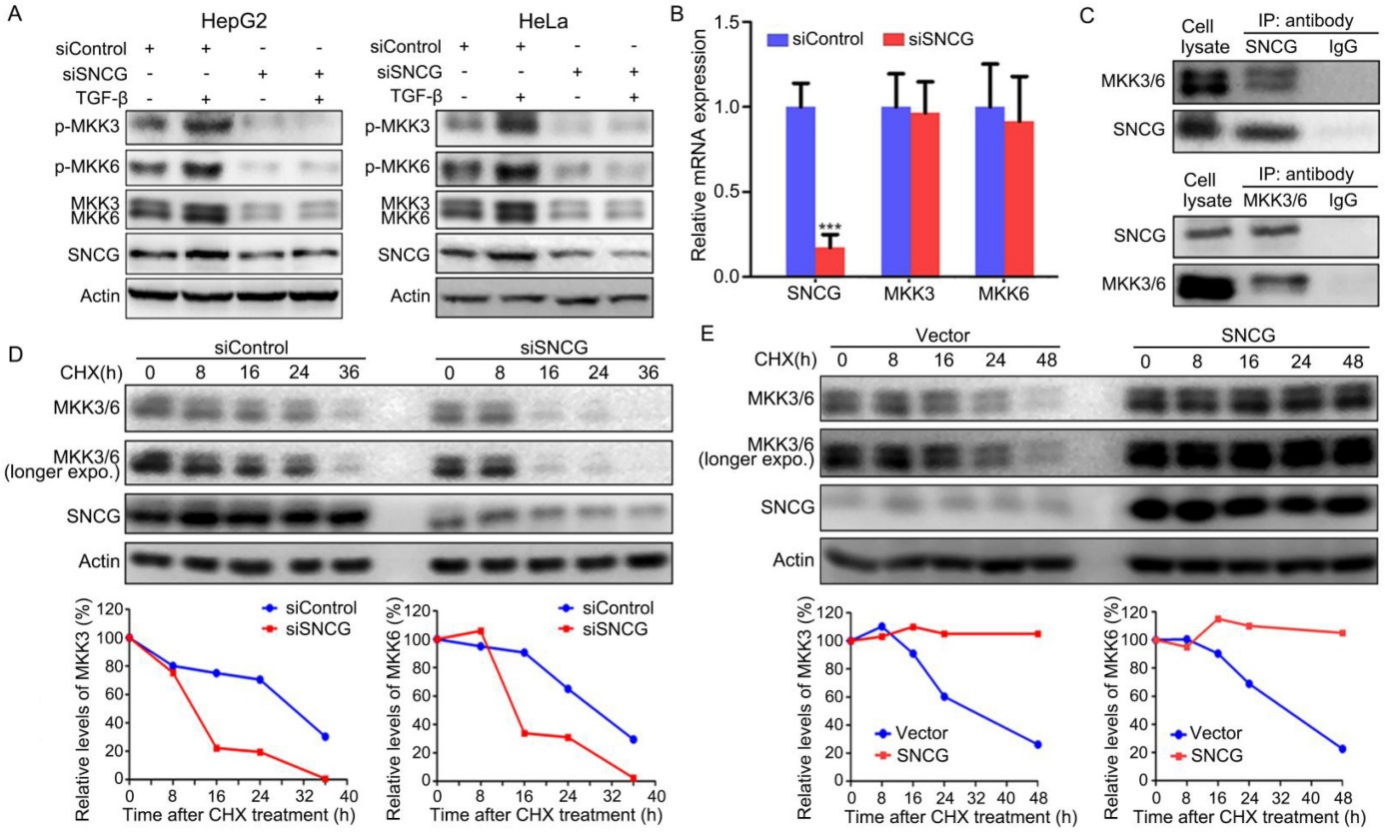
** SNCG stabilizes MKK3/6 and promotes TGF-β-induced p38MAPK phopshorylation. (A)** HepG2 and HeLa cells were transfected with siControl or siSNCG. Twenty-four hours later, the cells were treated with 5 ng/ml TGF-β for another 48h, followed by western blot analysis of indicated proteins.** (B)** HepG2 cells were transfected with 50 nM of siControl or siSNCG for 24h, followed by qRT-PCR analysis of *SNCG*, *MKK3* and *MKK6* transcription. The levels of transcripts in siControl-transfected cells were set as 1. Values represent mean ± SD. (*n* = 3). ***, *p* <0.001, compared with siControl-transfected cells. **(C)** HepG2 lysates were subjected to immunoprecipitation with anti-SNCG, anti-MKK3/6 or normal IgG, followed by western blot analysis of MEK3/6 and SNCG. **(D)** HepG2 cells were transfected with siControl or siSNCG. 24h later, the cells were treated with protein translation inhibitor cycloheximide (CHX, 100 μg/mL) for indicated periods, followed by western blot analysis of MKK3/6 and SNCG. The relative levels of MKK3/6 were plotted. For both siControl- and siSNCG-transfected cells, the levels of MKK3/6 at 0 h were set as 100%. **(E)** HepG2 cells were transfected with the empty vector or SNCG expression plasmid, and treated with CHX for indicated periods, followed by western blot analysis of MKK3/MKK6 and SNCG. The relative levels of MKK3/6 were plotted. For both vector- and SNCG-transfected cells, the levels of MKK3/6 at 0 h were set as 100%.

While TGF-β-induced activation of TAK1-TRAF6-TAB1 is responsible for p38MAPK, Erk and JNK pnosphorylation, MKK3/6, MKK1 and MKK4 mediate the induction of p38MAPK, Erk and JNK phosphorylation by TGF-β-TAK1 signaling, respectively. Since SNCG knockdown predominantly suppresses TGF-β-induced p38MAPK phosphorylation, we then detected whether SNCG could regulate MKK3/6. Indeed, SNCG knockdown led to a decrease in the levels of total MKK3/6 and abrogated the induction of MKK3/6 phosphorylation by TGF-β in both HepG2 and HeLa cells (Fig. 2A). However, SNCG knockdown did not affect the levels of *MKK3/6* transcripts, suggesting that SNCG may affect MKK3/6 at protein level (Fig. 2B).

Next, we detected whether SNCG physically interacted with MKK3/6. To this end, HepG2 cell lysates were subjected to immunoprecipitation with anti-SNCG antibody, followed by western blot analysis of SNCG and MKK3/6. Immunoprecipitation of SNCG co-precipitated both MKK3 and MKK6, and vice versa (Fig. 2C). Meanwhile, SNCG did not interact with TAK1, TRAF6 and p38MAPK (Fig. S2). To determine whether SNCG affects the stability of MKK3/6 proteins, HepG2 cells were transfected with control siRNA or SNCG siRNA, followed by treatment with cycloheximide to inhibit protein synthesis and chasing the levels of MKK3/6 for different periods. After treating with cycloheximide, the levels of MKK3/6 proteins dropped more rapidly in SNCG-knockdown cells than that in non-knockdown cells (Fig. 2D). In contrast, overexpression of SNCG stabilized MKK3/6 proteins (Fig. 2E). Moreover, heat shock resulted in an increase in SNCG expression and modest decrease in the levels of MKK3 in HepG2 cells, while it did not affect MKK6 (Fig. S3). SNCG knockdown slightly accelerated the decrease in MKK3 levels following heat shock (Fig. S3).

### SNCG regulates MKK3/6 ubiquitination and proteasomal degradation

**Figure 3:**
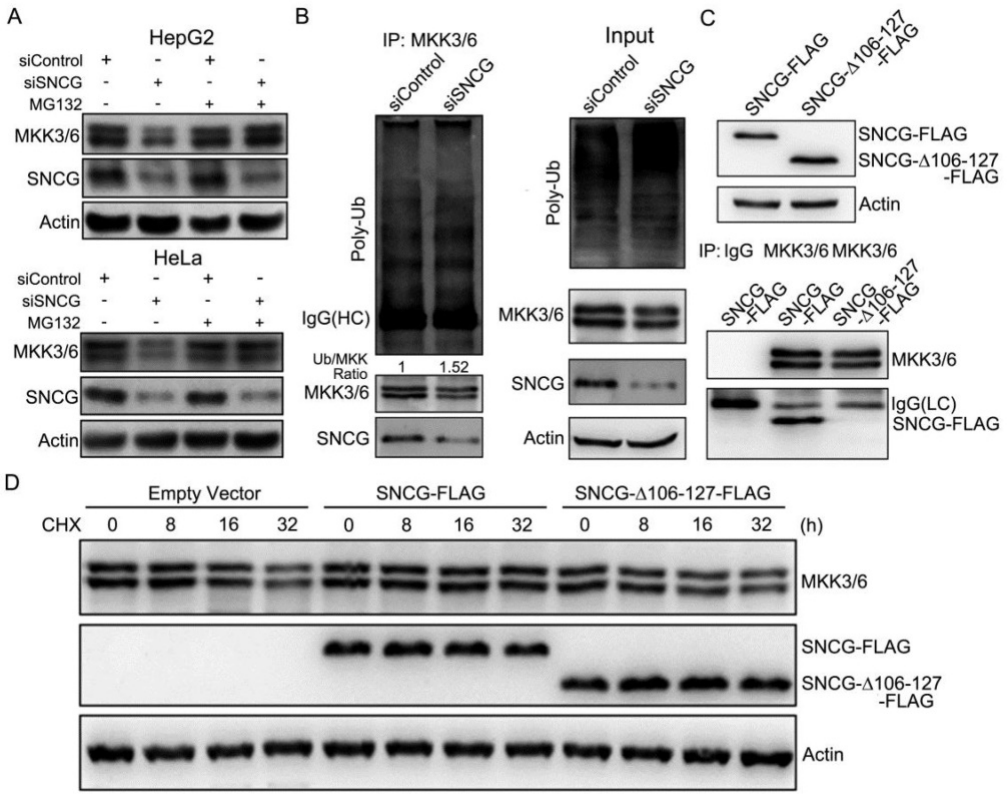
** SNCG silencing promotes MKK3/6 ubiquitination and proteasomal degradation. (A)** HepG2 and HeLa cells were transfected with siControl or siSNCG. 24h later, the cells were treated with or without proteasome inhibitor MG132 (5 μM) for another 48 h, followed by western blot analysis of MKK3/6 and SNCG.** (B)** HepG2 cells were transfected with siControl or siSNCG. Cell lysates were subject to immunoprecipitation with the MKK3/6 antibody, followed by western blot analysis of MKK3/6, SNCG and ubiuqitinated MKK3/6 (poly-Ub). HC, heavy chain. The indicated proteins in cell lysate were also shown (input). **(C)** The FLAG-tagged full-length SNCG (SNCG-FLAG) and C-terminal-truncated SNCG (SNCG_△106-127_-FLAG) were overexpressed in HepG2 cells, and validated by western blot analysis. The MKK3/6 immunoprecipitates were subject to western blot analysis of MKK3/6 and FLAG-tagged SNCG. LC, light chain. **(D)** HepG2 cells were transfected with empty vector, SNCG-FLAG and SNCG_△106-127_-FLAG, followed by treatment with CHX for indicated periods. The levels of MKK3/6 and FLAG-tagged SNCG were detected by western blotting.

To determine whether SNCG regulates proteasomal degradation of MKK3/6, HepG2 and HeLa cells were transfected with or without siSNCG, and treated with or without the proteasome inhibitor MG132, followed by western blot analysis of MKK3/6 levels. Treatment with MG132 abrogated the down-regulation of MKK3/6 expression by SNCG knockdown in both HepG2 and HeLa cells (Fig. 3A). Knockdown of SNCG by another set of siRNA consistently inhibited MKK3/6 expression, which was also reversed by MG132 (Fig. S4). Collectively, these data suggest that SNCG may inhibit proteasomal degradation of MKK3/6.

Furthermore, immunoprecipitation of MKK3/6 protein demonstrated that the relative levels of MKK3/6 ubiquitination were increased by SNCG knockdown (Fig. 3B). SNCG usually interacts with its targets via the C-terminal region of SNCG 30. We then prepared a FLAG-tagged construct for the expression of C-terminal-truncated SNCG (SNCG_△106-127_-FLAG) (Fig. 3C). Immunoprecipitation of MKK3/6 co-precipitated FLAG-tagged SNCG but not SNCG_△106-127_, indicating that the C-terminal region of SNCG is required for interaction with MKK3/6 (Fig. 3C). While overexpression of the full-length SNCG stabilized MKK3/6, overexpression of the C-terminal-truncated SNCG did not affect MKK3/6 stability (Fig. 3D), indicating that the C-terminal region of SNCG is critical for stabilizing MKK3/6.

### SNCG promotes cell migration and invasion through p38MAPK

**Figure 4:**
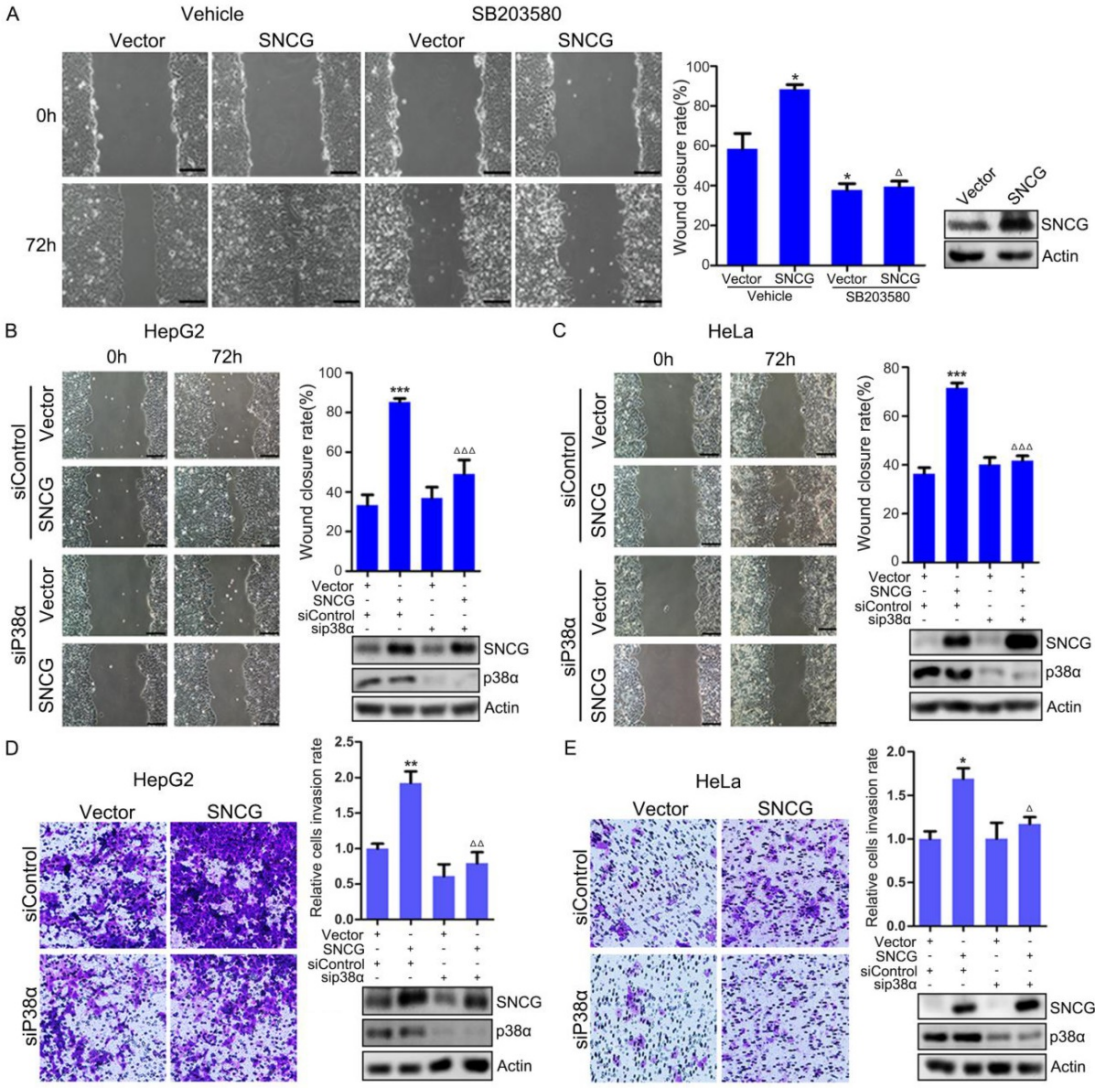
** SNCG promotes cell migration and invasion through p38MAPK. (A)** HepG2 cells were transfected with 1 μg/ml of the empty vector or SNCG expression plasmid, and then treated with or without 50 μM of SB203580 for another 24h, followed by wound healing assays. The relative wound closure rate was plotted. Values represent mean ± SD (*n* = 3). *, *p* < 0.05. **, *p* <0.01. ***, *p* <0.001. In parallel, the efficiency of SNCG overexpresion was detected by western blot analysis. **(B-C)** HepG2 or HeLa cells were transfected with the empty vector or SNCG plasmid, and then transfected with siControl or siP38α, followed by wound healing assays. Values represent mean ± SD (*n* = 3). *, *p* < 0.05. **, *p* <0.01. ***, *p* <0.001. In parallel, the efficiency of SNCG overexpresion and p38α knockdown was detected by western blot analysis. **(D-E)** HepG2 or HeLa cells were transfected with the empty vector or SNCG plasmid, and then transfected with siControl or siP38α, followed by cell invasion assays. The relative cell invasion rate was plotted. Values represent mean ± SD (*n* = 3). The invasion rate in vehicle-treated and vector-transfected cells was set as 1. *, *p* < 0.05. **, *p* <0.01. ***, *p* <0.001, compared with vehicle-treated and vector-transfected cells. In parallel, the efficiency of SNCG overexpresion and p38α knockdown was detected by western blot analysis.

To determine whether p38MAPK contributes to TGF-β-induced cell migration, HepG2 cells were treated with or without TGF-β and the p38MAPK inhibitor SB203580, followed by wound healing assays. Treatment with TGF-β promoted HepG2 cells migration (Fig. S5). SB203580 not only inhibited HepG2 cells migration, but also abrogated the promotion of cell migration by TGF-β (Fig. S5). In addition, HepG2 cells were transfected with the empty vector or SNCG-expressing plasmid, and treated with or without SB203580, followed by wound-healing assays. Overexpression of SNCG promoted HepG2 cells migration (Fig. 4A). SB203580 abrogated the promotion of cell migration by SNCG (Fig. 4A). Moreover, p38α knockdown abrogated the promotion of cell migration by SNCG in both HepG2 and HeLa cells (Figs. 4B and C).

Next, we determined whether SNCG could promote cell invasion through p38MAPK. To this end, HepG2 cells were transfected with or without SNCG expression plasmid and p38α siRNA, followed by detecting cell invasion in matrigeal-coated transwell chambers. Overexpression of SNCG promoted HepG2 cells invasion (Fig. 4D). p38α knockdown abrogated the promotion of HepG2 cells invasion by SNCG (Fig. 4D). Similar effects were detected in HeLa cells (Fig. 4E). Collectively, these data indicate that p38MAPK mediates the promotion of cancer cells migration and invasion by SNCG.

### p38MAPK mediates the promotion of MMP-9 expression by SNCG

**Figure 5:**
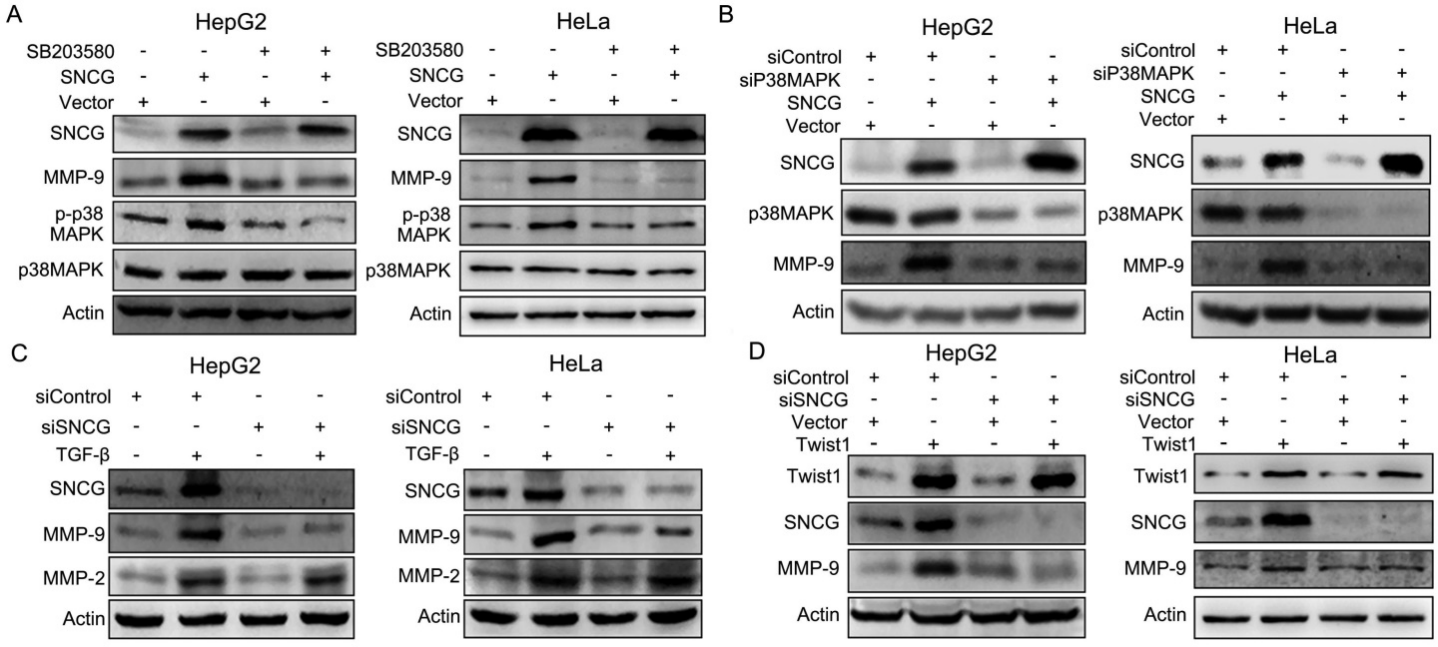
** SNCG promotes MMP-9 expression through p38MAPK. (A)** HepG2 and HeLa cells were transfected with 1 μg/ml of the empty vector or SNCG expression plasmid, and then treated with or without 50 μM of SB203580 for another 48 h, followed by western blot analysis of indicated proteins.** (B)** HepG2 and HeLa cells were transfected with 1 μg/ml of the empty vector or SNCG expression plasmid, and then transfected with siControl or siP38α, followed by western blot analysis of indicated proteins.** (C)** HepG2 and HeLa cells were transfected with 50 nM of siControl or siSNCG. 24h later, the cells were treated with or without 5 ng/ml of TGF-β for another 48h, followed by western blot analysis of indicated proteins.** (D)** HepG2 and HeLa cells were transfected with 1 μg/ml of the empty vector or Twist1 expression plasmid, and then transfected with siControl or siSNCG, followed by western blot analysis of indicated proteins.

Previous studies indicate that p38MAPK may promote cancer cell invasion through up-regulating MMP-9 27. We then detected whether SNCG up-regulated MMP-9 through p38MAPK. Indeed, overexpression of SNCG in HepG2 and HeLa cells induced p38MAPK phosphorylation and MMP-9 expression (Fig. 5A). Treatment with p38MAPK inhibitor abrogated the induction of MMP-9 by SNCG (Fig. 5A). Consistently, p38α knockdown abrogated the induction of MMP-9 by SNCG in both HepG2 and HeLa cells (Fig. 5B). In addition, SNCG knockdown suppressed the induction of MMP-9 by TGF-β in both HepG2 and HeLa cells, while the closely related MMP-2 was not affected by SNCG silencing (Fig. 5C). Knockdown of SNCG by another set of siRNA consistently abrogated the induction of MMP-9 by TGF-β (Fig. S6). Since Twist1 mediates the induction of SNCG expression by TGF-β, we also detected the role of SNCG in the induction of MMP-9 expression by Twist1. Overexpression of Twist1 in HepG2 and HeLa cells up-regulated both SNCG and MMP-9 expression. SNCG knockdown abrogated the induction of MMP-9 by Twist1 (Fig. 5D).

Since EMT is involved in TGF-β-induced cell migration and invasion, we then evaluated whether SNCG might promote EMT**.** While TGF-β induced the expression of EMT markers including fibronectin, N-cadherin and vimentin, SNCG knockdown had no effects on TGF-β-induced expression of these key EMT markers, suggesting that SNCG was not involved in TGF-β-induced EMT (Fig. S7).

### Inhibition of p38MAPK or MMP-9 abrogates the promotion of cancer cell invasion by SNCG

**Figure 6:**
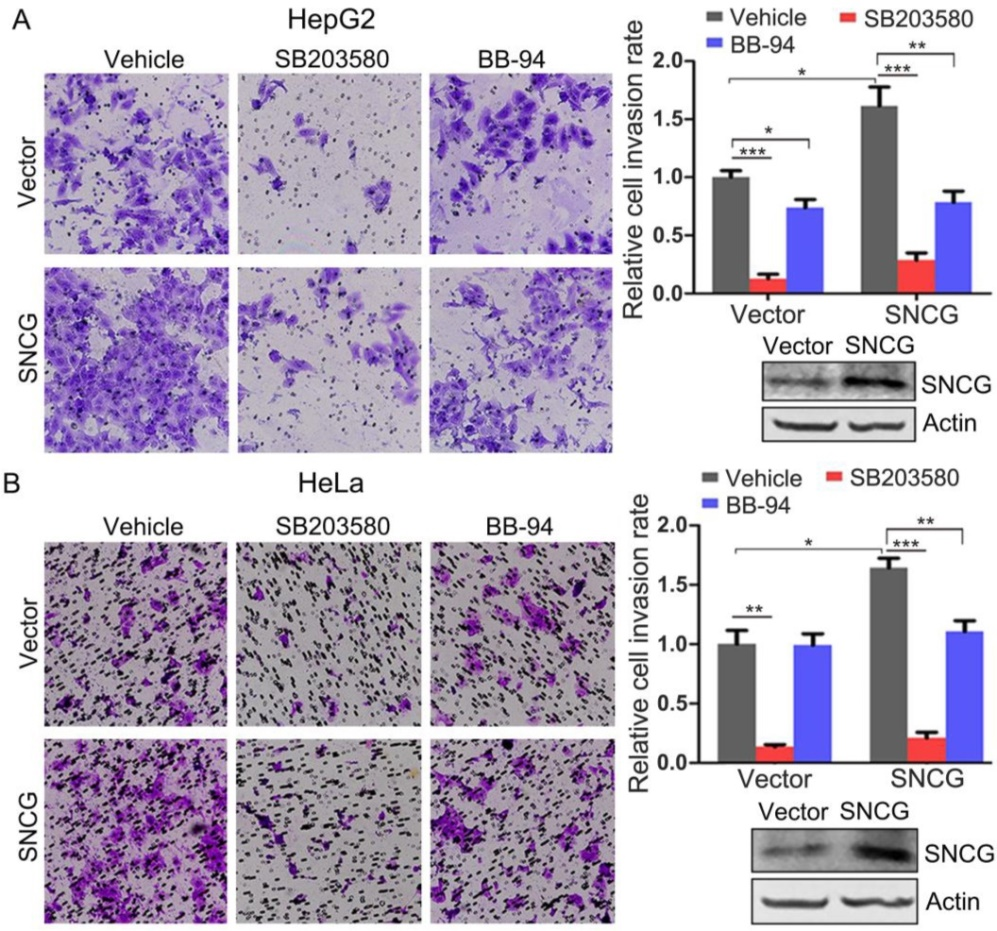
** Inhibition of p38MAPK or MMP abrogates the promotion of cell invasion by SNCG. (A)** HepG2 cells were transfected with 1 μg/ml of the empty vector or SNCG expression plasmid, and then treated with or without 50 μM of SB203580 and 10 μM of BB-94 for another 24h, followed by cell invasion assays. The relative cell invasion rate was plotted. The invasion rate in vehicle-treated and vector-transfected cells was set as 1. **(B)** HeLa cells were transfected with the empty vector or SNCG expression plasmid, and then treated with or without 50 μM of SB203580 and 10 μM of BB-94 for another 24h, followed by cell invasion assays. The relative cell invasion rate was plotted. Values represent mean ± SD (*n* = 3). *, *p* < 0.05;**,* p* <0.01; ***, *p* <0.001. In parallel, the efficiency of SNCG overexpresion was detected by western blotting.

The above-described data demonstrate that p38MAPK and MMP-9 mediate the promotion of cancer cells invasion by SNCG, suggesting that pharmacological inhibition of p38MAPK or MMP-9 may be potential approach for antagonizing the promotion of cancer cell invasion by SNCG. Indeed, treatment with a well-validated p38MAPK inhibitor SB203580 not only inhibited HepG2 and HeLa cells invasion, but also suppressed the promotion of cells invasion by SNCG. Similarly, treatment of HepG2 cells with the MMP inhibitor BB-94 also inhibited cells invasion, albeit to a lesser extent. BB-94 abrogated the promotion of HepG2 and HeLa cells invasion by SNCG (Fig. 6). Together, these data suggest that p38MAPK inhibitor may be promising agent to counteract SNCG-stimulated cancer cells invasion.

### p38MAPK inhibition abrogates the promotion of HCC metastasis by SNCG

**Figure 7:**
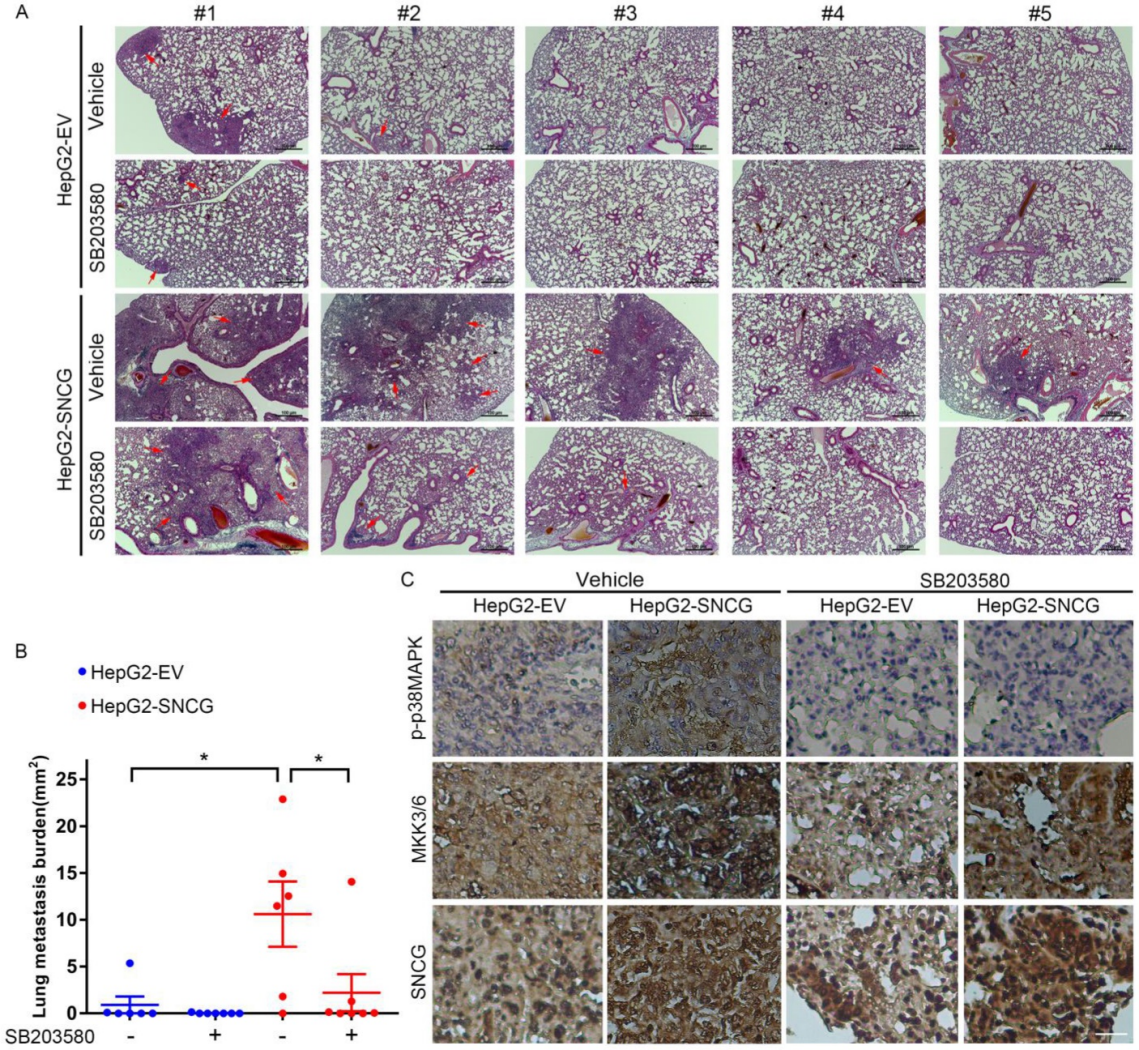
** Pharmacological inhibition of p38MAPK abrogates the promotion of lung metastasis by SNCG. (A)** HepG2 cells stably transfected with empty vector (EV) or SNCG expression plasmid were injected into the mice tail veins, and then treated with or without SB203580 daily. The mice were sacrificed 42 days after injection. The sections from paraffin-embedded lung tissues were subjected to HE staining. The red arrows-indicated metastatic foci in representative lung sections were shown. *Bars,* 100 μm. **(B)** The area of metastic foci in lung sections was measured**.** The lung metastasis burden in each group was plotted. *Bars,* SEM. *, *p* < 0.05. **(C)** The phosphorylation of p38MAPK and expression of MKK3/6 and SNCG in lung micrometastases were detected by immunohistochemistry. *Bar,* 25 μm.

To determine the effect of SNCG on HCC metastasis and the metastasis-suppressing effect of p38MAPK inhibitor, we proceeded to test the *in vivo* effect of SNCG overexpression and SB203580 in an experimental metastasis model. Forty days after tail vein injection of HepG2 cells stably transfected with empty vector (HepG2-EV) or SNCG expression plasmid (HepG2-SNCG) into nude mice, the lung metastasis of HepG2 cells was evaluated. Although there were no obvious nodules in the lung surface of all mice, microscopic analysis of hematoxylin-eosin-stained lung sections demonstrated that there were diffuse- or micro-metastasis in two of six mice injected with HepG2-EV cells, one of seven mice injected with HepG2-EV cells and treated with SB203580, and all mice injected with HepG2-SNCG cells (Figs. 7A and B). Overexpression of SNCG induced larger or more metastatic lesions in the lungs (Figs. 7A and B). Treatment with SB203580 suppressed the promotion of pulmonary metastasis by SNCG (Figs. 7A and B). Immunohistochemical analysis demonstrated that MKK3/6 expression and p38MAPK phosphorylation were increased in HepG2-SNCG micro-metastases compared with HepG2-EV (Fig. 3C). SB203580 inhibited p38MAPK phosphorylation in both HepG2-EV and HepG2-SNCG micro-metastases (Fig. 7C). Together, these data demonstrate that SNCG can promote the metastasis of HCC, and pharmacological inhibition of p38MAPK is an approach to suppressing the pro-metastasis effect of SNCG.

## Discussion

SNCG is an oncogene that can be induced by both IGF and TGF-β, which are critically involved in tumor progression 12, 28, 29. It is well-defined that TGF-β is a contextual tumor suppressor or promoter, depending on the tumor types and stages. Whereas TGF-β may inhibit tumor cell growth at the early stage, it usually promotes cancer metastasis at late stages. As a TGF-β- and IGF-responsive gene, SNCG is more frequently overexpressed in advanced cancers and involved in the stabilization or activation of some oncogenes such as IGF-IR and Akt, which have critical roles in tumorigenesis and drug resistance 2, 28, 30, 31. Preclinical studies have demonstrated that SNCG can promote cancer metastasis 14, 20, 21, 24. Hence, SNCG may be one of the mediators of TGF-β- or IGF-induced cancer metastasis.

TGF-β signaling is mediated by multiple pathways, including SMAD and non-SMAD pathways. While SMAD proteins directly mediate the genomic response to TGF-β, the non-canonical TGF-β signaling is mediated by SMAD-independent protein kinases such as MAPKs and JNK 32. Previous studies have demonstrated that there is cross-talk between the SMAD and non-SMAD pathways. For example, TGF-β-induced activation of MAPKs and JNK may phosphorylate SMADs to promote the transcription of oncogenes 32. On the other hand, SMADs can mediate TGF-β-induced activation of MAPKs. Expression of dominant-negative SMAD3 and SMAD4 attenuates TGF-β-induced activation of p38MAPK and ERK1/2, respectively 33. While SNCG is up-regulated by TGF-β-SMAD-Twist1 axis 12, the current study demonstrates that it also stimulates p38MAPK signaling, a non-SMAD pathway in TGF-β signaling. Thus, SNCG may be a novel link between the SMAD and non-SMAD pathways in TGF-β signaling.

p38MAPK activation is directly catalysed by upstream MAPK kinases including MKK3 and MKK6. TGF-β-activated protein kinase 1 (TAK1) and TAK1-binding protein 1 work together to mediate TGF-β-induced MKK3/6-p38MAPK signaling 34. Our current study demonstrates that SNCG can prevent proteasomal degradation of MKK3/6 proteins. Whilst little is known about the regulation of MKK3 stability, previous study has demonstrated that the stability of MKK6 protein is regulated by FBXO31-mediated ubiquitination at Lys82 residue 35. Lys48-linked ubiquitination of MKK6 leads to proteasomal degradation 35. It remains to know whether other ubiquitin ligases may be involved in regulating MKK3/6 stability. SNCG appears to be a novel regulator of MKK3/6 stability, while it warrants further study to decipher the mechanism.

Previous studies have demonstrated that p38MAPK promotes the migration and invasion of variety types of human tumor cells 36, 37. Mechanistically, p38MAPK may activate MAPK-activated protein kinase 2, which in turn phosphorylates HSP27 and then promotes cytoskeletal remodelling, leading to increased cell motility and invasiveness 38, 39. In addition, p38MAPK may promote cancer metastasis by activating NF-*k*B and stabilizing FOXC1 40, 41. Moreover, MMPs have important roles in cell invasion and cancer metastasis 42. TGF-β also upregulates MMPs to promote cell invasion through extracellular matrix remodeling. One mechanism underlying the up-regulation of MMP-9 by TGF-β is dependent on p38MAPK 43. The up-regulation of MMP-9 by p38MAPK may involve transcription factors including AP1 and Sp1 44, 45. While TGF-β induces both MMP-2 and MMP-9 expression, SNCG knockdown only abrogates the induction of MMP-9 but not MMP-2. The promotion of MMP-9 expression by SNCG is dependent on p38MAPK. P38MAPK-mediated induction of MMP-9 expression may contribute, in part, to the promotion of cancer cell invasion by SNCG.

The prevention and treatment of cancer metastasis remain to be a great challenge. Pharmacological inhibition of p38MAPK may suppress the metastasis of breast cancer and melanoma 46, 47. The oral p38MAPK inhibitor ralimetinib (LY2228820) has undergone clinical trials and demonstrated acceptable tolerability 48. A phase 1b/2 clinical trial indicates that the addition of ralimetinib to gemcitabine-carboplatin regimen may moderately improve the progression-free survival in patients with recurrent platinum-sensitive ovarian carcinoma 49. Except for ralimetinib, there are other p38MAPK inhibitors under development, such as BIRB796 and its derivatives 50. Given that inhibition of p38MAPK may suppress the metastasis of SNCG-overexpressing cancer cells, targeting p38MAPK may be an approach for treating SNCG-positive cancers.

In summary, our findings indicate that SNCG is a regulator of the MKK3/6-p38MAPK pathway in TGF-β signaling. SNCG interacts with MKK3/6 and enhances their stability, thereby promoting TGF-β-induced p38MAPK activation, cell migration and invasion. Our finding that p38MAPK deficiency restricts SNCG-induced cell migration and invasion provides insights into mechanisms underlying the promotion of tumor progression by SNCG. Targeting p38MAPK may be considered for treating SNCG-overexpressed cancer.

## Supplementary Material

Supplementary figures and tables.

## Abbreviations

EMT: epithelial-mesenchymal transition
Erk: extracellular signal-activated kinase
JNK: Jun N-terminal kinase
MAPK: mitogen-activated protein kinase
MKK3/6: MAPK kinase 3/6
MMP: matrix metalloproteinase
PI3K: phosphoinositide 3-kinase
SMAD: mothers against decapentaplegic homolog
SNCG: gamma synuclein
TAK1: TGF-β-activated protein kinase 1
TGF-β: transforming growth factor-β
