# Supplementary material for: Gamma synuclein promotes cancer metastasis through the MKK3/6-p38MAPK cascade
Source: Int J Biol Sci. 2022 May 1;18(8):3167–77. doi: 10.7150/ijbs.69155 (PMC9134903; doi:10.7150/ijbs.69155)
Supplement: Supplementary file 1 — Supplementary figures and tables. [file ijbsv18p3167s1.pdf]

Supplementary Information

Gamma synuclein promotes cancer metastasis through a MKK3/6-p38MAPK cascade

Jieya Liu, Ting Shao, Jin Zhang, Qianyi Liu, Hui Hua, Hongying Zhang, Jiao Wang, Ting Luo, Yuenian Eric Shi, Yangfu Jiang

The supplementary information contains figure S1-8 and table S1-2.

Supplementary figures

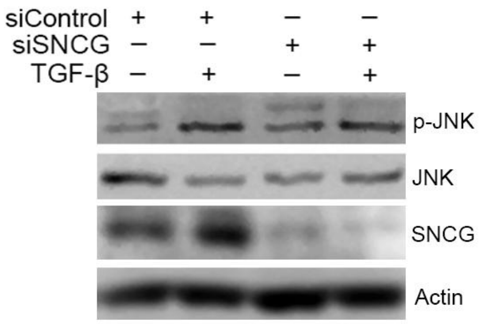

Figure S1. SNCG knockdown does not affect the induction of JNK phosphorylation by TGF-β. HepG2 cells were transfected with siControl or siSNCG. 24 hours later, the cells were treated with 5 ng/ml TGF-β for another 48h, followed by Western blot analysis of indicated proteins.

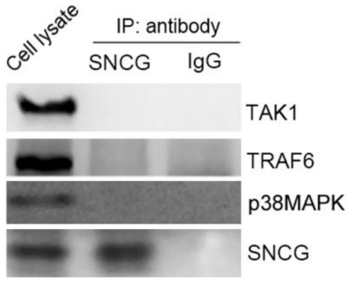

Figure S2. SNCG does not interact with p38MAPK, TRAF6 and TAK1. HepG2 lysates were subjected to immunoprecipitation with SNCG antibody or normal IgG, followed by Western blot analysis of p38MAPK, TRAF6, TAK1 and SNCG.

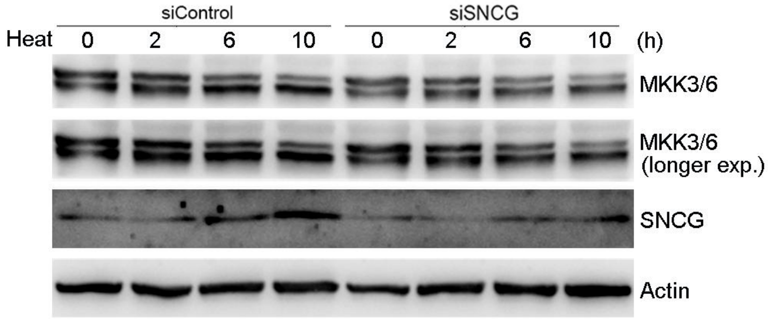

Figure S3. SNCG knockdown accelerates heat shock-induced decrease in MKK3 levels. HepG2 cells were transfected with siControl or siSNCG, followed by incubation at 42℃ at indicated periods. The total proteins were harvested and subject to western blot analysis of indicated proteins.

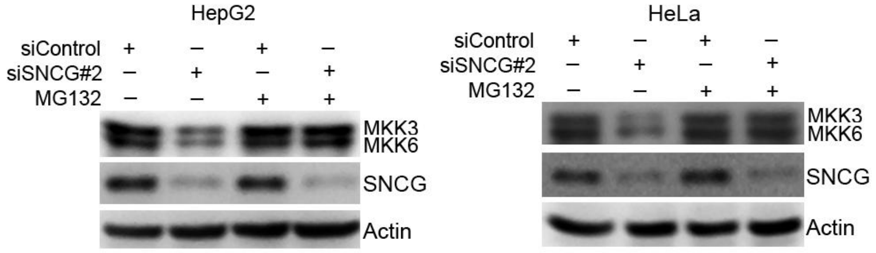

Figure S4. MG132 abrogates MKK3/6 down-regulation resulting from SNCG knockdown. HepG2 and HeLa S3 cells were transfected with siControl or siSNCG#2. 24h later, the cells were treated with or without proteasome inhibitor MG132 (5 μM) for another 48 h, followed by western blot analysis of MEK3/6 and SNCG.

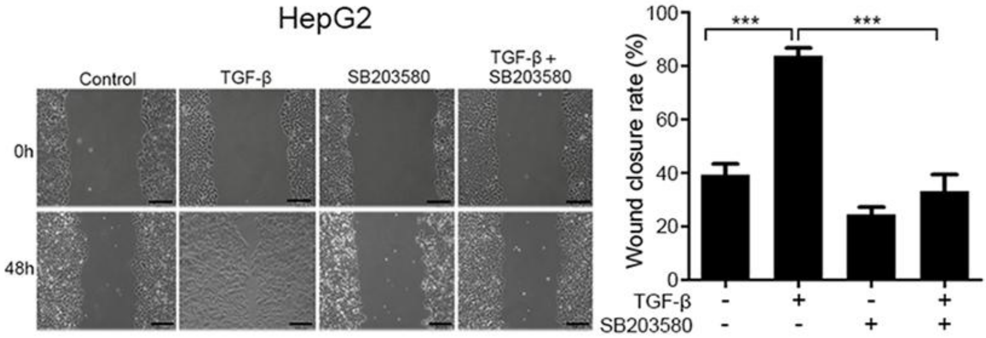

Figure S5. SB203580 abrogates TGF-β-induced HepG2 cell migration. HepG2 cells were treated with or without 50 μM of SB203580, 5 ng/ml of TGF-β and 2 μg/ml of mitomycin. *Scale bar,* 200 μm. The wound closure rate was shown. Values represent mean ± SD (n = 6). ***, p < 0.001.

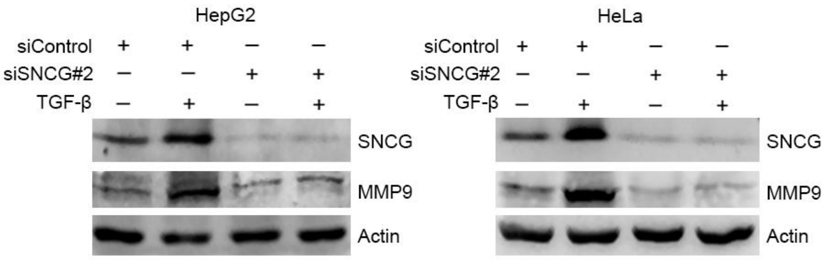

Figure S6. SNCG knockdown abrogates TGF-β-induced MMP-9 expression. HepG2 and HeLa S3 cells were transfected with siControl or siSNCG#2. 24 hours later, the cells were treated with 10 ng/ml of TGF-β for another 48h, followed by Western blot analysis of indicated proteins.

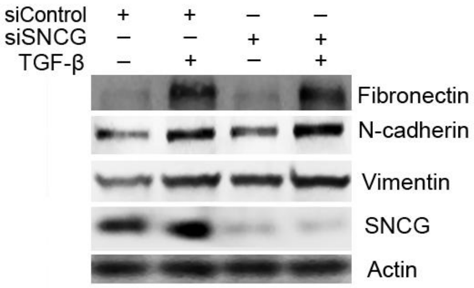

Figure S7. SNCG knockdown has no effects on TGF-β-induced fibronectin, vimentin and N-cadherin expression. HepG2 cells were transfected with siControl or siSNCG. 24 hours later, the cells were treated with 5 ng/ml of TGF-β for another 48h, followed by Western blot analysis of indicated proteins.

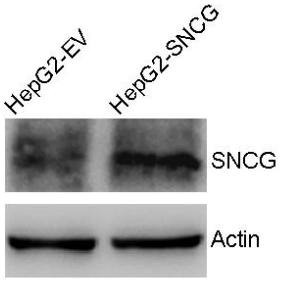

Figure S8. The expression of SNCG in HepG2 cells transfected with the empty vector (HepG2-EV) or SNCG expression plasmid (HepG2-SNCG). HepG2-EV and HepG2-SNCG cells lysates were subjected to Western blot analysis of indicated proteins.

Supplementary tables

Table S1: siRNA target sequences

siRNA	Target sequences
siSNCG	5′-GCAGCTGAGAAGACCAAGG-3′
siSNCG#2	5′-GGAGAATGTTGTACAGAGC-3′
siP38α	5′-CAGACCATTTCAGTCCATCATTCAT-3′

Table S2: Primers sequences

Gene	Forward sequences	Reverse sequences
SNCG	5′-ATGCGGCTGCCCACGCTCCT-3′	5′-GTCTTGGCTCCCACATACAT-3′
MKK3	5′-CTTGGTGACCATCTCAGAACTGG-3′	5′-CTTCTGCTCCTGTGAGTTCACG-3′
MKK6	5′-GCCTCAGACCAGTTCCAC-3′	5′-GCATCTTCTCCACCACCC-3′
GAPDH	5′-AATCGCATCATCATAACCTG-3′	5′-CATCCTGCCCATCATACTC-3′
